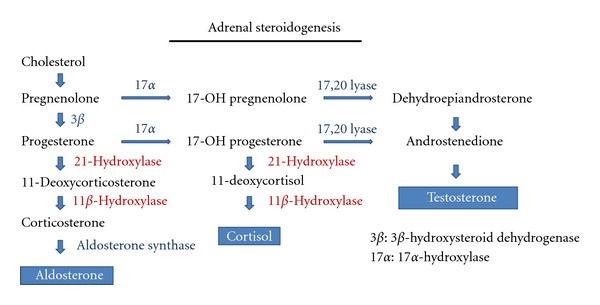# Adrenal Incidentalomas with Supraphysiologic Response to ACTH Stimulus: A Case Report

**DOI:** 10.1155/2012/503290

**Published:** 2012-10-14

**Authors:** Marianna Antonopoulou, Asya Perelstein

**Affiliations:** ^1^SUNY Downstate Medical Center, 450 Clarkson Avenue, Box 1205, Brooklyn, NY 11203, USA; ^2^VA Medical Center, 800 Poly Place, New York, NY 11209, USA

## Abstract

We present the diagnostic approach of a patient with adrenal incidentalomas. A 72-year-old African American male had a CT scan of the abdomen showing right and left adrenal masses measuring 5 × 3.5 cm and 3.7 × 2.9 cm, respectively. The patient had negative hormonal workup. The radiologist insisted that the CT findings are consistent with adrenal hyperplasia, and therefore he underwent ACTH stimulation to rule out late-onset congenital adrenal hyperplasia (CAH). The stimulation test revealed that 17-hydroxyprogesterone and 11-deoxycortisol increased to levels high enough to confirm CAH, but cortisol had exaggerated response as well, thus making the diagnosis of CAH unlikely where metabolism is shifted to precursors. Subsequently, the patient underwent screening for Cushing's syndrome (CS) with a dexamethasone suppression test. Patient failed the suppresion test, raising the issue for subclinical CS (SCS), likely due to ACTH-independent macronodular adrenal hyperplasia. Our patient had been diagnosed with MGUS and so far there are only 3 case reports of extramedullary plasmacytoma arising from the adrenals. One was bilateral and one had functional abnormalities. Our differential diagnosis includes subclinical CS with aberrant receptors versus a functioning extramedullary plasmacytoma.

## 1. Introduction

Adrenal incidentalomas are very common nowadays, and subclinical autonomous cortisol hypersecretion is the most frequent hormonal abnormality and it may be intermittent. This is why there is a need for periodic hormonal and morphological evaluation for several years [1]. Also, there is evidence that patients with subclinical Cushing's syndrome (SCS) have increased cardiovascular risk profile, similar to that described in overt Cushing's syndrome (CS), making the diagnosis of SCS of great importance [2]. We present the diagnostic approach of a patient with bilateral adrenal incidentalomas. 

## 2. Case Report

### 2.1. History of Present Illness

**Figure 1:**
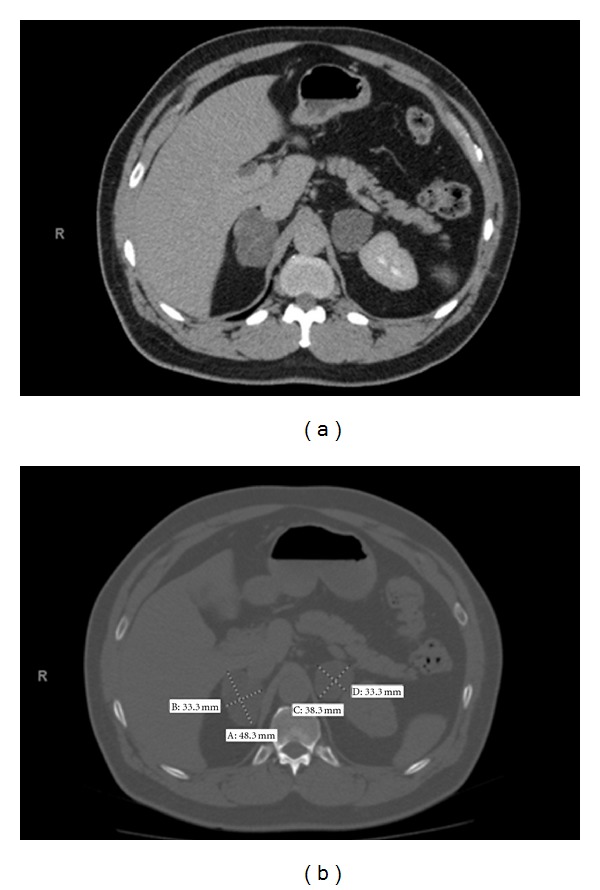
CT scan of abdomen/pelvis with and without contrast as seen during follow-up imaging: lipid-rich adrenal masses bilaterally, right adrenal mass 4.8 × 3.3 cm, previously measured approximately 5.0 × 3.5 cm. Left adrenal mass is 3.8 × 3.3 cm, previously measured 3.7 × 2.9 cm.

The patient is a 72-year-old African-American male. After complaining of chronic back pain in 9/2009, he had a spine MRI that revealed arachnoiditis and prominence of bilateral adrenals. CT scan of abdomen/pelvis was done to further evaluate the adrenals that revealed lipid-rich adrenal masses bilaterally, right adrenal mass 5 × 3.5 cm, and left adrenal 3.7 × 2.9 cm (Figure 1). Both the masses were less than 20 Hounsfield units after contrast injection.

 Besides his chronic back pain, the patient had no other complaints. He was very adherent to his medication although his blood pressure on occasion was ranging from 150/78 to 160/96. His medications include diltiazem 240 mg daily, lisinopril 10 mg daily, omeprazole 20 mg, aspirin 81 mg, plavix 75 mg, and tramadol 50 mg as needed for pain.

### 2.2. Past Medical History

It was hypertension, hyperlipidemia, spinal stenosis, monoclonal gammopathy of uncertain significance (MGUS), and coronary artery disease. 

### 2.3. Social History

Patient was never a smoker. He rarely drank a glass of wine at social events and denied any use of illicit drugs or over-the-counter medication. 

### 2.4. Family History

It was breast cancer and colon cancer in one sister and his father, respectively. His mother died of stroke and father of colon cancer. 

### 2.5. Hospital Course

After the CT scan results, the patient was sent to our endocrine clinic for evaluation. On physical exam, the patient has no cushingoid features. He is slightly overweight, with BMI of 26 and no evidence of abnormal skin pigmentation, striae or evidence of proximal muscle weakness. He was screened for depression as part of the standard of care, and the patient health questionnaire (PHQ-9) was negative. He denied obstructive symptoms at night and admits waking up in the morning refreshed from the night sleep. Patient underwent hormonal workup to rule out hormone-producing adrenal tumors three times, including 24-hour urine cortisol level, 24-hour urine metanephrines, serum aldosterone, and plasma renin activity (at that time patient was not on lisinopril), all of which were normal. During followup, CT scans showed the adrenal masses remained stable in size, but the radiologist insisted that the CT findings are consistent with adrenal hyperplasia. In view of the patient also being hypertensive, he underwent an ACTH (cosyntropin) stimulation test to rule out late-onset CAH (congenital adrenal hyperplasia). In our case the patient's cortisol went from baseline of 419 mmol/L to 1,848.53, 17-hydroxyprogesterone from 0.33 nmol/L to 11.69, and 11-deoxycortisol from 0.5772 mg/L to 10.97. ACTH at baseline was 1.034 pmol/L. CAH is unlikely in this case, since one would expect insufficient production of cortisol and a shift towards the precursors. Our patient had exaggerated response of both cortisol and the precursors. The patient subsequently underwent overnight 1 mg dexamethasone suppression test as screening for CS and also low dose (4 mg) suppression test. Patient failed both suppresion tests with morning cortisol level of 215.2 mmol/L and 204.16 mmol/L, respectively. Normal response should be morning cortisol less than 49.662 mmol/L, according to the 2008 Endocrine Society Guidelines. The above results were consistent with SCS, most likely secondary to aberrant receptors. Thereafter, patient underwent workup to rule out the ectopic expression of glucose-dependent insulinotropic peptide (GIP) receptor in zona fasciculata cells.

The postdexamethasone suppression test baseline fasting cortisol level was 215 mmol/L and 2-hours after a mixed meal was 201, essentially ruling out GIP-dependent CS. The full workup for aberrant receptors could not be performed because TRH and GnRH are not available in the USA and the patient started developing chest discomfort, had a positive stress test, and underwent coronary artery catheterization with stenting. The patient is currently recovering well.

## 3. Discussion

ACTH (adrenocorticotropic hormone) stimulation test is traditionally used in the diagnosis of adrenal insufficiency and also to diagnose cases of CAH. In CAH where 21-hydroxylase or 11-beta hydroxylase deficiency is common, the ACTH stimulation shifts the steroidogenesis to the precursors (Figure 2). 

So 17-hydroxyprogesterone concentrations range typically from 10 to 100 nmol/L. Cortisol in healthy individuals should double from baseline and usually ranges from 386.26 to 689.75 mmol/L.

Abnormal response to ACTH stimulation test has been shown in patients with major depression [3]. Also, dysregulation of cortisol production is seen in patients with obstructive sleep apnea, obese patients, or with significant alcohol intake [4–7]. All of the above were excluded in our patient. Also, after careful review of the patient's medication list, no medication was identified that would justify this response.

As supported by the literature search, supraphysiologic response of cortisol to ACTH stimulus occurs with ACTH-independent CS, also known as ACTH-independent macronodular adrenal hyperplasia (AIMAH). It is ACTH independent because endogenous ACTH levels are low or normal, and cortisol secretion is regulated by hormones other than ACTH via aberrant expression of hormone receptors, like *β*-adrenergic, vasopressin, LH, FSH, TSH, and GIP receptors in the adrenals.

AIMAH accounts for <1% of overt CS. It usually presents in the 5th-6th decade of life as subclinical CS. It can be sporadic or familial (autosomal dominant). Supraphysiologic response of cortisol to ACTH occurs with AIMAH because the ACTH receptor gene remains expressed. However, there is also relatively inefficient hormonal synthesis with decreased expression of steroidogenic enzymes. The hormone secretion in AIMAH results from an increase in the number of adrenocortical cells rather than an augmented synthesis per cell and this is why steroid precursors may be found elevated [8–13]. 

Diagnosis of subclinical CS is very challenging and currently there are no guidelines. However, according to some authors, the presence of at least two of the following: abnormal 1 mg dexamethasone suppression test, elevated 24 hr urine cortisol, ACTH below 2.2 pmol/L, constitutes diagnosis [14]. The adrenal glands are usually nodular but cases without nodules have also been described [15].

Unfortunately, the workup for aberrant receptors, to confirm ACTH-independent macronodular adrenal hyperplasia, is very tedious and some of the suggested stimuli like TRH or GnRH are not commercially available in the USA. Thus, we were unable to perform the protocol on our patient, but the workup for subclinical Cushing's syndrome was positive and the abnormal response to ACTH stimulation test is suggestive of our diagnosis. The identification of aberrant receptors provides new opportunities in pharmacological therapy as an alternative to adrenalectomy. The patient had negative workup for GIP receptor, but in view of his CAD, we can give a trial of b-blocker in case his aberrant receptors are beta-adrenergic [16]. There is still no consensus for the management of subclinical CS. However, according to one small study it appeared that surgical adrenalectomy was more beneficial than medical management as it improved the possible metabolic consequences like diabetes and hypertension, which are important risk factors for increased morbidity and mortality [2, 17].

Another consideration arises because of the patient's previous diagnosis of MGUS. Thus far, in the literature there have been 3 cases of extramedullary plasmacytoma arising from the adrenals. One was actually bilateral and one was a functioning tumor secreting catecholamines [18–20]. Of course in our patient the tumor size has been stable for the past 2 years and urine was negative for Bence Jones protein, leading us away from a diagnosis of malignancy. The only way though to reach a certain diagnosis is through histologic examination. Unfortunately in this case, the patient's comorbidities and recent cardiac procedure defer surgery, which would have been diagnostic and curative in the case of either functioning plasmacytoma or ACTH-independent adrenal hyperplasia with subclinical CS. It lies in the clinician's judgment to make those difficult decisions.

## 4. Conclusions

Subclinical Cushing's syndrome can be a cause of increased morbidity and mortality and the physician should be vigilant to diagnose and treat when appropriate. Since there is no consensus currently it relies on the clinician's judgment how to manage these challenging cases.

## Figures and Tables

**Figure 2:**